# Traditional or adaptive design of experiments? A pilot-scale comparison on wood delignification

**DOI:** 10.1016/j.heliyon.2024.e24484

**Published:** 2024-01-11

**Authors:** Hannu Rummukainen, Hanna Hörhammer, Pirkko Kuusela, Jorma Kilpi, Jari Sirviö, Mikko Mäkelä

**Affiliations:** VTT Technical Research Centre of Finland Ltd., PO Box 1000, 02044 VTT Espoo, Finland

**Keywords:** Active learning, Bayesian optimization, Machine learning, Response surface methodology, Wood delignification, Alkaline oxidation

## Abstract

Traditional design of experiments and response surface methodology are widely used in engineering and process development. Bayesian optimization is an alternative machine learning approach that adaptively selects successive experimental conditions based on a predefined performance measure. Here we compared the two approaches using simulations and empirical experiments on alkaline wood delignification to identify important benefits and drawbacks of Bayesian optimization in the context of design of experiments. The simulations showed that the selection of initial experiments and measurement noise influenced the convergence of the Bayesian optimization algorithm to known optimal conditions. Both methods, however, showed comparable pilot-scale results on optimal digestion conditions, where high cellulose yields were combined with acceptable kappa numbers and pulp viscosities. Bayesian optimization did not enable a decrease in the number of experiments required for reaching these conditions but provided a more accurate model in the vicinity of the optimum based on additional modelling and cross-validation. These results shed light on the practical differences between the two methodologies for process development and are an important contribution to the chemometrics and machine learning communities.

## Graphical abstract

**Figure:**
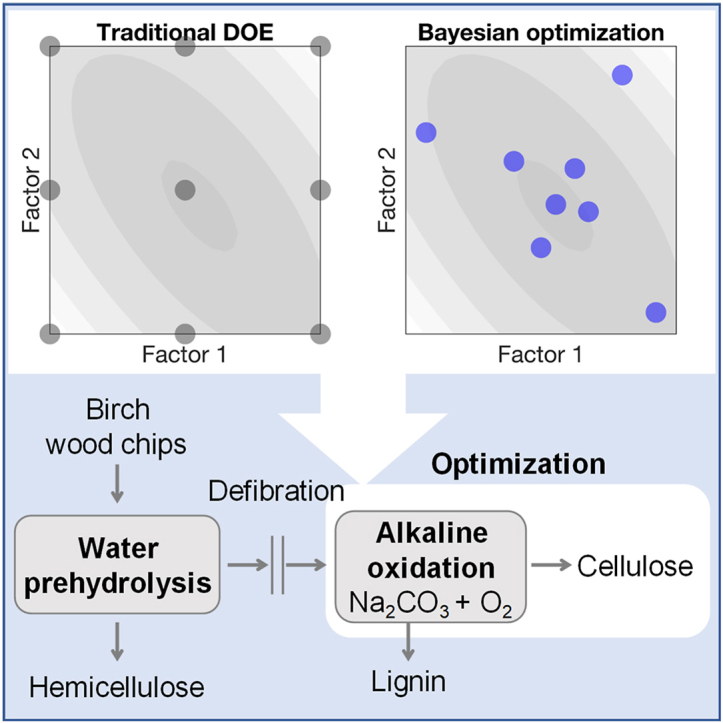

## 1 Introduction

Design of experiments and response surface methodology are widely used in engineering and are considered as the gold standard for optimization in industrial experiments. The principles for describing response surfaces were originally established by chemists and statisticians [1,2] and have largely remained the same since the 1950s. These traditional methods have recently been complemented with machine learning alternatives based on Bayesian optimization [3,4]. Bayesian optimization combines adaptive sampling with non-parametric modelling to identify candidates for the next experiments based on expected improvement and uncertainty within an experimental domain [[3], [4], [5]]. This active learning strategy combines experimental design and optimization into a single process and can potentially reduce the number of experiments required to estimate optimal experimental conditions. Promising applications have recently been reported from materials science [6,7], process development [[8], [9], [10], [11]], and life sciences [12].

Bayesian optimization methods provide an adaptive alternative for optimization tasks, as each new experiment uses information from previous experiments by fitting a probabilistic model on the available data. This probabilistic model is typically a Gaussian random field defined by its mean and covariance functions, whose parameters are selected by maximizing the posterior likelihood of the observed data. The posterior likelihood depends on the prior likelihoods assigned to different values of the model parameters, but the influence of the prior likelihoods diminishes as more experimental data is acquired. The relevant performance indicators are expressed within an explicit objective function, which is actively used for choosing the experimental conditions. Each new experiment is determined to balance exploitation and exploration, i.e., seeking an optimal value of the objective function based on the current probabilistic model (exploitation) and reducing uncertainty by acquiring more experimental data in conditions that are distant from the earlier experiments (exploration). Bayesian optimization algorithms can be shown to converge to the globally optimal objective value even in the presence of zero-mean measurement errors under some mild theoretical assumptions [5].

Traditional design of experiments and Bayesian optimization can both be used for optimizing empirical systems where exact physical or chemical equations of the underlying phenomena are either not known or do not work properly under non-ideal conditions. The potential benefits of Bayesian optimization raise important questions from the perspective of design of experiments. For example, can this active learning strategy lead to a significant decrease in the number of experiments required to reach optimal process conditions within a given application? And what are the most important benefits and drawbacks that should be considered when choosing between the two methodologies? Systematic studies comparing these approaches are currently lacking and are important to evaluate their suitability for a wide range of optimization tasks.

Here, we compared traditional design of experiments and Bayesian optimization using simulations and empirical experiments. We first simulated laboratory measurements using an existing dataset on carbohydrate extraction from wood to evaluate how the number of initial experiments and measurement noise influenced Bayesian optimization. We then compared the two methods using pilot-scale experiments on alkaline wood delignification. Linear regression models were determined separately for cellulose yield and properties based on a Box Behnken design and the modelling results were compared with Bayesian optimization in the three-dimensional search space. The results shed light on the practical differences between the two methods for process development and are an important contribution to the chemometrics and machine learning communities.

## 2 Materials and methods

### 2.1 Simulations

Experiments were simulated based on previous research on carbohydrate extraction from wood [13]. The dataset is given in Table A.1 (see Appendix) and describes experiments where extraction temperature, time, and liquid-to-solid ratio were varied, and carbohydrate yield was determined from the extraction liquid. The experiments were organized according to a central composite face-centered design on three factors with three experiments in the design center. The aim was to maximize yield, which was predicted as a function of the extraction conditions using a linear regression model.

The linear regression model was used to simulate experimental results in Bayesian optimization. We simulated 3000 sequences of 20 experiments where we varied the number of initial experiments and the share of random measurement noise. We restricted the available initial experiments to the unique experimental settings in the dataset (rows 1–15 in Table A.1) and chose four to eight initial experiments to maximize G-efficiency [14] within the available experimental locations by assuming a linear main effect model. The remaining experiments were then selected adaptively by using the Bayesian optimization algorithm. Three levels of random measurement noise were generated by drawing noise samples defined by 0–0.5 % points in standard deviation from a zero-mean normal distribution and were added to predicted yield given by the linear regression model. Since the results of the simulation runs depended on random numbers, each sequence of 20 experiments was repeated 200 times to estimate the variances of the outcomes. These 200 replicates were split evenly between the different G-optimal design alternatives, which were available for each number of initial experiments. In total 60,000 artificial experiments were simulated across five choices in the number of initial experiments, three levels of random measurement noise, and 200 repetitions of the sequences of 20 experiments.

### 2.2 Experiments

**Fig. 1:**
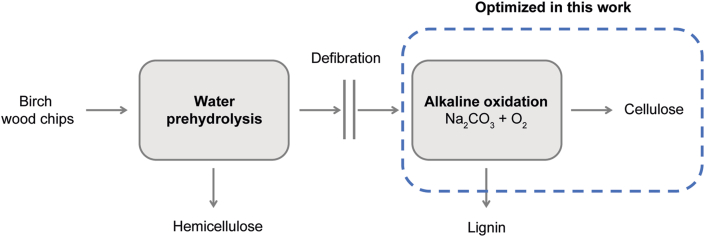
A schematic illustration of the wood delignification process.

Birch wood was then empirically delignified with alkali under pressurized oxygen. The experiments were performed in pilot conditions where 2.5 kg of wood chips were first pretreated in 15 L rotating reactors with 10 L of water at 170 °C for 2 h. The pretreated chips were washed with water and mechanically defibrated with a disc refiner. A batch of 1 kg of the remaining wood material was oxidized under pressure in a 43 L reactor in a 2 mol kg^−1^ Na_2_CO_3_ solution with 100 rpm mixing. After the reactions the pulps were washed with water and screened to separate rejects. Gravimetric yield, kappa number (ISO 302 [15]), viscosity (ISO 5351 [16]), and alkali resistance (ISO 699 [17]) were determined from the screened pulps using standard methods in the field. A schematic illustration of the delignification process is shown in Fig. 1.

The individual experiments were first organized according to a Box Behnken design [18] where digestion temperature, time, and liquid-to-solid ratio were varied on three different levels. The design included three experiments in the design center and required a total of 15 experiments. The design range, i.e., the minimum and maximum values for the conditions, was defined based on our previous experience [19,20] on alkaline delignification with the aim of maximizing cellulose yield with defined targets for kappa number and pulp viscosity. Five experiments included in the Box Behnken design were then used as starting experiments for Bayesian optimization and ten new experiments were performed in conditions suggested by the optimization algorithm. A total of 25 pilot experiments were performed across the two methods. The defined digestion conditions, chosen responses, and their target ranges are summarized in Table 1.

**Table 1:** Digestion conditions and chosen responses during the experiments.

	Parameter	Symbol	Design or target range
Digestion conditions	Temperature	T	120–160 °C
	Time	τ	60–180 min
	Liquid-to-solid ratio	R	5–10
Responses	Cellulose yield	Y	to be maximized
	Kappa number	Κ	0–6.57
	Pulp viscosity	η	400–600 mL/g

### 2.3 Data analyses

Cellulose yield in the pulps was determined after the hemicellulose and lignin contents had been subtracted from the screened pulp mass. Kappa number was used as an estimate for pulp lignin content. The lignin content was estimated by dividing the determined kappa number with a conversion factor of 6.57 [21]. Although the exact relation depends on wood species and delignification conditions [15], we did not expect that potential error in the applied conversion factor would have influenced the conclusions of this study. Alkali resistance describes hemicellulose content, which was estimated by subtracting alkali resistance from 100 %. Linear regression models were then determined separately for cellulose yield, kappa number and pulp viscosity following standard methodology in the design of experiments field [[22], [23], [24], [25], [26]]. In short, the digestion conditions were coded so that they ranged from −1 to 1 in coded units and second order regression models were determined as functions of the digestion conditions. For *k* variables a second order regression model can be expressed as, Eq. (1):

(1) $$y=\beta_{0}+\sum_{i=1}^{k}\beta_{i}x_{i}+\sum_{i=1}^{k-1}\sum_{j=i+1}^{k}\beta_{ij}x_{i}x_{j}+\sum_{i=1}^{k}\beta_{ii}x_{i}^{2}+\varepsilon$$

where y denotes the values of a response, $\beta_{0}$ the average value of y in the design center, $\beta_{i}$, $\beta_{ij}$, and $\beta_{ii}$ the first order, interaction and second order model coefficients, respectively, $x_{i}$ the coded variable levels and ε the model residual. Statistical significance of the coefficients was determined by testing them against zero using a *t*-test with the residual degrees of freedom. Statistically insignificant coefficients (p > 0.10) were removed iteratively one by one unless they were included in significant interactions or higher order model terms. We point out that although Eq. (1) is a quadratic function, the model is still linear in the unknown parameters β and we hence refer to it as a linear regression model [22,23].

The objective function in the Bayesian method expresses the desired process outcomes in mathematical terms. The objective function was defined based on the three responses, Eq. (2):

(2) $$g\left(Y,Κ,\eta \right)=Y-w_{1}\;\max\left(0,Κ-6.57\right)-w_{2}\;\max\left(0,Κ-10\right)-w_{3}\;\max\left(0,400-\eta \right)-w_{4}\;\max\left(0,\eta -600\right)$$

where g denoted the scalar objective as a function of cellulose yield Y, kappa number Κ and pulp viscosity η. Penalty terms were assigned to kappa number and pulp viscosity if they were outside their target ranges (Table 1) and the weights were set as $w_{1}$ = 0.2, $w_{2}$ = 1.8, $w_{3}$ = 1.0, and $w_{4}$ = 0.1, Eq. (2). A corner of the three-dimensional search space was ruled out a priori as it was considered unlikely to lead to acceptable results. This restriction eliminated 1/48 of the volume of the search space and was formulated as an explicit constraint, Eq. (3):

(3) $$\frac{T-120}{20}+\frac{\tau -60}{60}+\frac{R-5}{2.5}\geq 1$$

where T, τ, and R denoted digestion temperature, time, and liquid-to-solid ratio, respectively.

The same Bayesian optimization method was used for the simulations and the performed pilot-scale experiments. The objective function and its uncertainty were estimated using a Gaussian process regression model with a Matérn-5/2 kernel [27]. Gaussian measurement noise was assumed to be homoscedastic and maximum a posteriori estimates were used for the length scale and noise parameters. The prior distributions for the length scale and noise parameters were equivalent to the ones used in the simulations; see the Appendix for details. During the first nine iterations the next experimental conditions were selected by maximizing the *noisy expected improvement* acquisition function [28,29]. Noisy expected improvement was computed as a Monte-Carlo approximation of the expectation, Eq. (4):

(4) $$a_{\text{NEI}}\left(x\right)=E_{g_{\max}}\int_{g_{\max}\;}^{\infty }f_{x}\left(y\right)\left(y-g_{\max}\right)\;dy$$

where $a_{\text{NEI}}\left(x\right)$ denoted the noisy expected improvement acquisition function at the hypothetical next experiment x, $g_{\max}$ the maximum true objective value reached so far, and $f_{x}\left(y\right)$ the Gaussian density function representing the objective value uncertainty at x. The expectation was determined over the distribution of true objective values at the previous experiment conditions according to the current Gaussian process regression model. On the final tenth experimental iteration, the experiment conditions were selected by maximizing the posterior mean of the Gaussian process regression model.

Leave-one-out cross-validation was used to quantitatively compare the experimental results of the linear regression model on cellulose yield with a Gaussian process regression model on cellulose yield. To make the models directly comparable, the Gaussian process regression model was computed for cellulose yield without any penalty terms, instead of the full objective function. Otherwise, the parameters of the Gaussian process regression model were estimated in the exact same way as in the Bayesian optimization method. Both models were trained using the data from only fifteen experiments, but the resulting root mean squared errors were determined based on all experimental locations and two subsets of locations, one subset defined by the target ranges of pulp viscosity and kappa number indicated in Table 1, and the other by vicinity to the best process conditions found by either method. The data analyses for the linear models were performed in Matlab® (The MathWorks, Inc.) and the Bayesian optimization was implemented in Python using the BoTorch library [29].

## 3 Results and discussion

### 3.1 Simulations

We used simulations to evaluate how the number of initial experiments and measurement noise influenced simulated yield during Bayesian optimization. A total of 60,000 simulated experiments were performed using an existing dataset on carbohydrate extraction from wood and the aim was to determine the number of iterations required to reach a known optimum under different conditions. The regression model for predicting yield is shown in Eq. (A.1). Derivation of the model equation with a constant liquid-to-solid ratio indicated a predicted yield maximum of 12.7 % with the extraction conditions 172 °C, 97 min and a liquid-to-solid ratio of 6. This was considered as the optimum in the simulation experiments, although the true optimum yield may have differed.

**Fig. 2:**
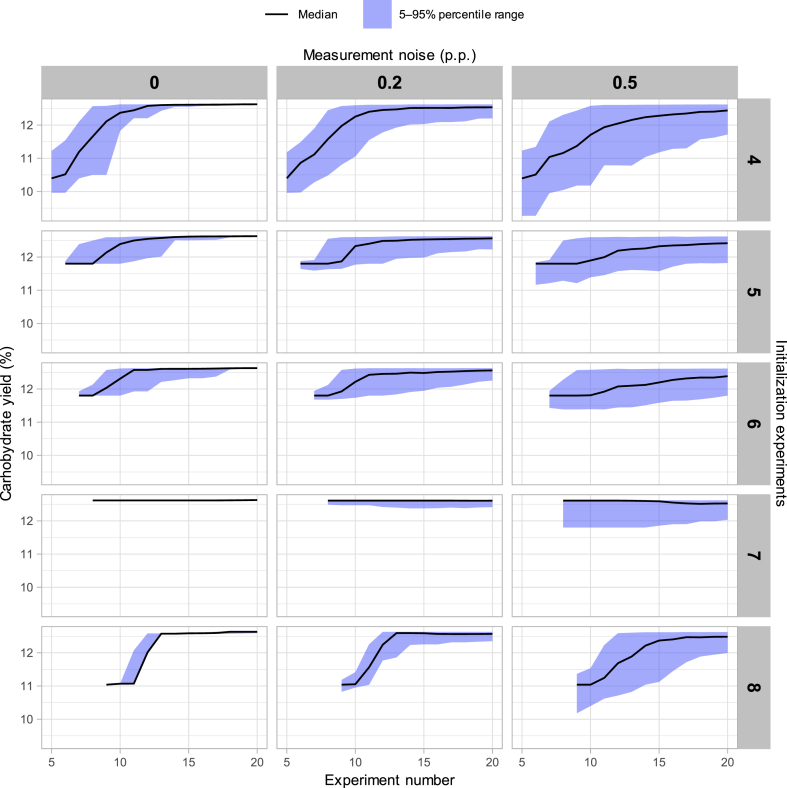
Distributions of the Bayesian optimization results from the simulated experiments. The column headers on top indicate the levels of measurement noise, and the row headers on the right indicate the number of initialization experiments. The horizontal axes in each subgraph show the number of simulated experiments and the vertical axes show the highest carbohydrate yields reached so far without measurement error.

Most of the experimental sequences simulated with Bayesian optimization showed steady improvements in predicted yield but also indicated that the number and selection of initialization experiments influenced the convergence of the Bayesian optimization algorithm to a known optimum (Fig. 2). The designs for each number of initialization experiments were chosen to maximize G-efficiency to safeguard against worst-case predictions by minimizing maximum prediction errors within the available sampling locations [14]. As shown in Fig. 2, the predicted yields improved early in the 20 experiment sequences using 5–7 initial experiments compared to the other alternatives.

The experimental sequences simulated with five or six initial experiments included a center-point experiment with a predicted yield of 11.8 % and the sequences with seven initial experiments included an experiment with a nearly optimal yield of 12.6 %. The final alternative with eight initial experiments included only the extreme corners of the design space as a two-level factorial design with three variables is G-optimal for a main effect model. These results showed that choosing promising initialization experiments had a significant effect on the convergence of the Bayesian optimization algorithm. Higher levels of measurement error slowed down convergence and required more experiments to reach a known optimum while generating a larger variance across the outcomes (Fig. 2). Although Bayesian optimization would in theory find the global optimum with even larger measurement errors, noisier experiments increased the uncertainties in choosing the next experiments and therefore increased the number of required iterations. Overall, we observed that the effects of initialization were small over 15–20 experiments, but choosing promising initial experiments improved results in shorter experimental sequences.

### 3.2 Experiments

We then performed empirical experiments to compare traditional design of experiments with Bayesian optimization. Birch wood was delignified in pilot-scale conditions with the aim of maximizing cellulose yield with predefined target ranges for kappa number and pulp viscosity (Table 1). The experiments were first organized according to a Box Behnken design, which is an efficient alternative for a three-level design. The results are shown in Table A.2. Cellulose yield, kappa number and pulp viscosity were then modelled separately using multiple linear regression. The final model equations are given in Eqs. (A.2)-(A.4) and the results are summarized in Table 2.

**Table 2:** Linear regression models determined based on the Box Behnken design.

Response	Transformation	Degrees of freedom		R^2^	R^2^_pred_
		Total corrected	Model		
Cellulose yield (%)	None	13	5	0.90	0.73
Kappa number	log_10_	14	3	0.95	0.91
Viscosity (mL g^−1^)	None	14	4	0.97	0.92

The final linear models explained 90–97 % of the variation in the determined responses (Table 2). One observation was excluded from the model for cellulose yield. As shown in Table A.2, experiment number 6 showed the lowest cellulose yield and was clearly an outlier based on externally studentized model residuals [30]. This difference was likely caused by an incomplete recovery of the delignified pulp as the experiment also resulted in a considerably lower screened pulp yield than the other experiments. The exclusion increased the R^2^ and R^2^_pred_ values of the cellulose yield model from 0.83 and 0.58 to 0.90 and 0.73, respectively. The R^2^_pred_ values of the three models were in the range 0.73–0.92 and indicated that the models for kappa number and pulp viscosity were the most reliable ones for predicting new observations based on cross-validation (Table 2). Cellulose yield was determined based on screened pulp yield, hemicellulose content, and kappa number and uncertainties in these analytical quantities were likely reflected in the reliability of the final model.

**Fig. 3:**
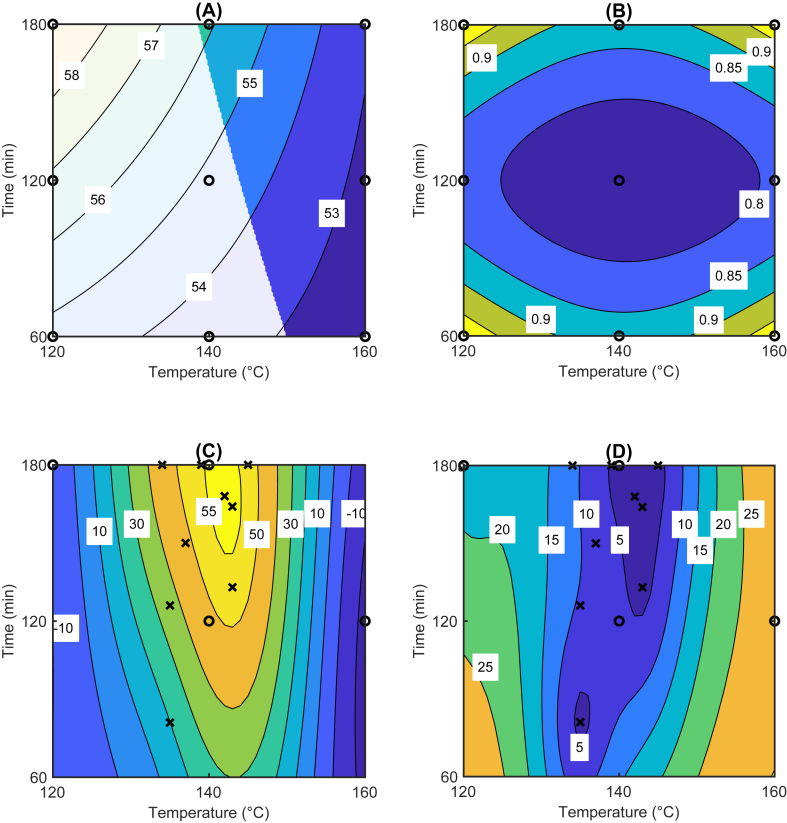
Predicted cellulose yield (%) constrained by the defined target ranges for kappa number and pulp viscosity based on the Box Behnken design (A) and the respective standard error of prediction (B) with a liquid-to-solid ratio of 10. Predicted objective function (unitless) based on Bayesian optimization after 10 iterations (C), and the respective standard error of prediction (D). The symbols show the sampling locations, which were projected to the plane across the design space. Symbols ‘o’ denote initial experiments and ‘×’ denote experiments suggested by the Bayesian optimization algorithm.

Estimated cellulose yield and properties can be presented as response surfaces, which provide an intuitive tool to visualize the model predictions as a function of the delignification conditions. We present these response surfaces as two-dimensional plots based on delignification temperature and time by setting liquid-to-solid ratio at its maximum value, which provided the highest predicted cellulose yields. The results for the three models are shown in Fig. A.1. In Fig. 3A we have further superimposed these contours to show predicted cellulose yield constrained by the defined target ranges for kappa number and pulp viscosity. The results indicated that cellulose yield was maximized at moderate temperatures and long reaction times based on the predictions (Fig. 3A). Lower reaction temperatures increased cellulose yield but resulted in higher kappa numbers and pulp viscosities exceeding the defined targets for cellulose properties. Higher kappa and pulp viscosity suggested that these reaction conditions were not sufficient to reach the qualities required for dissolving pulp. Increased processing temperature and time enhance the degradation of wood, which resulted in decreased pulp yield and decreased lignin content [31]. Dissolving grade pulp has high cellulose content and low content of other compounds, i.e., lignin [32], and the viscosity of dissolving pulps is in the range of 400–600 mL/g [33]. Higher alkali charges, higher processing temperature and longer retention times generally result in increased cellulose depolymerization and therefore decreased viscosity [34].

**Fig. 4:**
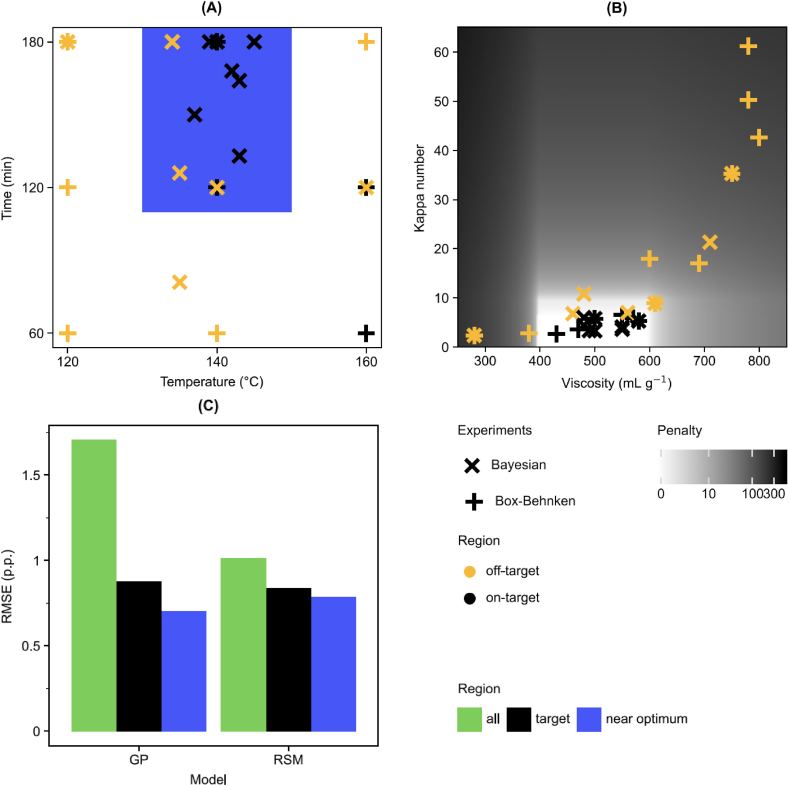
Bayesian optimization experiments (×) and traditional Box Behnken experiments (+) in terms of process conditions (A) and responses (B), and cross-validation results (C). In (A) and (C) the blue shading indicates the area near the optimum point at 140 °C, 160 min and liquid-to-solid ratio 10. In (B) the target region is on white background and the shading outside the target region shows the penalty in the Bayesian optimization objective function. The marker colors in (A) and (B) indicate whether responses are in the target region in (B). The cross-validation results (C) indicate the root mean square error of cellulose yield predictions for all points, for the points in the target region, and for the points near the optimum. (For interpretation of the references to color in this figure legend, the reader is referred to the Web version of this article.)

We chose five experiments to initialize the Bayesian optimization algorithm based on the simulation results described in Section 3.1. These five experiments were chosen a priori as a diverse selection of digestion conditions expected to generate variation in cellulose yield and properties. Ten subsequent experiments were then performed one by one at conditions suggested by the optimization algorithm. The experimental conditions and the numerical results from Bayesian optimization are described in Table A.3. The final predicted values of the objective function and the respective standard errors are shown in Fig. 3 and the experimental conditions together with the determined kappa number and viscosity values are visualized in Fig. 4.

The experimental conditions with the highest cellulose yield of 56.3 % had already been found during the first initialization experiment (Table A.3) and by the end of the experiments the Bayesian optimization method was converging towards these conditions. Values of the objective function generally increased with increasing iterations but did not surpass the first initialization experiment during the iterations. As shown in Fig. A.2Fig. A.2, the expected improvement values computed by the algorithm remained below 0.8, which suggested that the highest objective value from the first initialization experiment was nearly optimal. After the eighth experiment the modelled uncertainty on the optimum values was decreasing, but the expected improvement values were sufficiently high that we could not rule out the possibility of further yield increases of 0.5–1%, which would have been meaningful for the application. Overall, the optimization algorithm selected more experiments near the most promising conditions compared with the traditional Box Behnken design (Figs. 3 and 4A). The determined kappa numbers and pulp viscosities during the Bayesian experiments were also within or very close to their defined target ranges, see Fig. 4B. The respective cellulose yields were within the range 53–55%.

The Gaussian process regression model of the optimization objective function after fifteen experiments is shown in Fig. 3C and the uncertainty model is given in Fig. 3D. The objective function is not directly comparable with the response surface of predicted cellulose yield in Fig. 3A. Both models, however, indicated that the optimal digestion conditions were approximately reached with a temperature of 140 °C, a reaction time of 180 min, and a liquid-to-solid ratio of 10. The shape of the objective function response surface indicated that near-optimal results could also be achieved with somewhat shorter times in the range 150–180 min. Fig. 3D indicated that the uncertainty was lowest near the optimal digestion conditions where most of the experiments were performed. These findings were contrary to the traditional Box Behnken design, in which the lowest uncertainty was situated in the center of the design where the replicate experiments were performed. The increase in standard errors towards the edges of the design was however negligible and suggested that the traditional approach provided reliable predictions near the optimal digestion conditions.

One of the experiments in the Box Behnken design was identified as an outlier based on model residuals and was excluded from the cellulose yield model (Table 2). The Gaussian process regression in the Bayesian optimization method was performed automatically with no manual oversight and outliers could not be excluded. Since Bayesian optimization focused on experiments close to the optimum conditions, it would have been difficult to judge whether an experiment that was further away was a potential outlier. Bayesian optimization methods are also more complex than linear regression models, and their application involves choosing prior distributions, an acquisition function, and kernel functions for Gaussian process regression. The regression method in Bayesian optimization should consider, for example, prior knowledge on whether the response is monotonic or convex [35,36], which can affect the number of required experiments. More research is needed to identify proper variants, prior choices [37], and potential outliers in Bayesian optimization for design of experiments.

### 3.3 Comparison of regression models

Traditional response surface methodology aims to model the variation of the response accurately in the entire experimental design space. Bayesian optimization, however, aims to model the variation of the objective function more accurately in the regions where the value of the objective function is near optimal. A direct quantitative comparison of the results of the two methods was not straightforward since the objective function included multiple penalty terms. However, we used leave-one-out cross-validation as an attempt to compare the prediction errors of two cellulose yield models. These models included the linear regression model we had determined for cellulose yield based on the Box Behnken design (Table 2, Fig. 3), and another cellulose yield model we created for comparison purposes with the Gaussian process regression method used in Bayesian optimization.

The cross-validation results expressed as the root mean squared error of predicted cellulose yield are shown in Fig. 4C and the predicted vs. observed values are given in Fig. A.3Fig. A.3. Fig. 4C shows errors for all 25 experiments and two subsets of experiments. The *target* subset was defined a priori based on the target ranges defined in Table 1 and included twelve experiments. The *near optimum* subset included fourteen experiments which were chosen a posteriori by being close the optimum, specifically with digestion time over 110 min and temperature between 130 and 150 °C. The results showed that the traditional linear regression model generated lower cross-validation errors over the full design space, but the differences within the target region were small. The Gaussian process model showed lower cross-validation errors only in the immediate vicinity of the optimal conditions. The errors of the Gaussian process model increased in the target region due to two experiments with a digestion temperature of 160 °C shown in black in Fig. 4A. We note that neither of these two experiments were originally included in the experiments of the Bayesian optimization method and we had to extrapolate the Gaussian process model predictions at these locations to enable comparing the cross-validation errors within the target range. The cross-validation subsets, however, provided a general overview of the reliability of the two approaches within different parts of the design space.

## 4 Conclusions

We compared traditional design of experiments and response surface methodology with adaptive Bayesian optimization and used simulations to first evaluate how the number of initial experiments and measurement noise influenced convergence during Bayesian optimization. The simulations showed that the selection of initial experiments had a substantial effect on the progress of Bayesian optimization and that increasing levels of noise slowed down the convergence of the algorithm to a pre-defined optimum. The initialization alternatives influenced the optimization mainly on shorter experimental sequences and increasing levels of noise suggested that the algorithm effectively chose the experimental conditions at random. We advise using domain knowledge in the choice of the initial design. Traditional experimental designs are good alternatives to evaluate the general behavior of the response with noisy experiments in cases where this information is important.

The two methods were then compared using empirical pilot-scale experiments, which showed comparable results on optimal digestion conditions, where high cellulose yields were combined with acceptable kappa numbers and pulp viscosities. The Bayesian optimization method started approaching these conditions again towards the end of the experimental sequence and selected more experiments closer to the optimum. Bayesian optimization, however, did not enable a significant decrease in the number of experiments required for reaching the optimum in the three-dimensional search space based on our results. Model cross-validation showed that the Bayesian optimization method provided a more accurate model of cellulose yield near the optimal conditions while its cross-validation errors clearly increased under sub-optimal conditions. The key difference we emphasize is that the Bayesian optimization method requires a clearly defined performance indicator, which should be maximized or minimized, and there is no need to reliably describe the behavior of the response within the entire design range. In contrast, traditional experimental designs generally consider prediction errors across most of the design space and the results can be reused to evaluate potentially favorable process conditions based on alternative performance indicators.

The two methods, however, are not mutually exclusive. Design of experiments includes established tools which can be used to select initial experiments for Bayesian optimization. These tools include, for example, space-filling designs or parameters which aim to rank design alternatives by considering their predictive properties based on a preliminary model assumption as illustrated by our approach on G-efficiency. Multiple criteria on design optimality can be combined to pre-evaluate initial design candidates from an available set of sampling locations and are preferably combined with domain knowledge in formulating the preliminary model assumption.

## Ethics statement

Review and/or approval by an ethics committee was not needed for this study because the study did not involve interaction with or observation of people, use of personal data, or any kind of animals. Informed consent was not required for this study because the study did not involve any study participants or patients.

## Data availability statement

The data used in this research is contained in the Appendix, which is included as supplementary material.

## CRediT authorship contribution statement

**Hannu Rummukainen:** Writing – review & editing, Writing – original draft, Visualization, Validation, Software, Methodology, Formal analysis, Conceptualization. **Hanna Hörhammer:** Writing – review & editing, Validation, Resources, Methodology, Investigation, Data curation, Conceptualization. **Pirkko Kuusela:** Writing – review & editing, Conceptualization. **Jorma Kilpi:** Writing – review & editing, Visualization, Conceptualization. **Jari Sirviö:** Writing – review & editing, Methodology, Conceptualization. **Mikko Mäkelä:** Writing – review & editing, Writing – original draft, Visualization, Validation, Supervision, Software, Project administration, Methodology, Funding acquisition, Formal analysis, Conceptualization.

## Declaration of competing interest

The authors declare that they have no known competing financial interests or personal relationships that could have appeared to influence the work reported in this paper.
