# Supplementary material for: Traditional or adaptive design of experiments? A pilot-scale comparison on wood delignification
Source: Heliyon. 2024 Jan 11;10(2):e24484. doi: 10.1016/j.heliyon.2024.e24484 (PMC10826314; doi:10.1016/j.heliyon.2024.e24484)
Supplement: Multimedia component 1 [file mmc1.docx]

# Traditional or adaptive design of experiments? A pilot-scale comparison on wood delignification

Hannu Rummukainen^a*^, Hanna Hörhammer^a^, Pirkko Kuusela^a^, Jorma Kilpi^a^, Jari Sirviö^a^, Mikko Mäkelä^a^

^a^ VTT Technical Research Centre of Finland Ltd., PO Box 1000, 02044 VTT Espoo, Finland

* corresponding author, e-mail: [hannu.rummukainen@iki.fi](mailto:hannu.rummukainen@iki.fi)

A linear regression model was used to simulate experimental results for carbohydrate yield. The final regression model for carbohydrate yield in Table A.1 was, Eq. (A.1):

$\hat{y}_{c}=11.8+0.26x_{1}-0.17x_{2}+0.81x_{3}-3.99x_{1}x_{2}-3.32x_{1}^{2}-2.67x_{2}^{2}$ (A.1)

where $\hat{y}_{c}$ denoted predicted carbohydrate yield (%) and $x_{1}$, $x_{2}$, and $x_{3}$ the coded values for extraction temperature (^o^C), time (min) and liquid-to-solid ratio, respectively. The values of the coefficients have been rounded.

Table A.1: The carbohydrate extraction dataset.

| Experiment | Temperature (^o^C) | Time (min) | Liquid to solid -ratio | Carbohydrate  yield (%) |
| --- | --- | --- | --- | --- |
| 1 | 150 | 30 | 4 | 2.0 |
| 2 | 190 | 30 | 4 | 9.4 |
| 3 | 150 | 180 | 4 | 8.1 |
| 4 | 190 | 180 | 4 | 1.2 |
| 5 | 150 | 30 | 6 | 2.2 |
| 6 | 190 | 30 | 6 | 11.3 |
| 7 | 150 | 180 | 6 | 10.2 |
| 8 | 190 | 180 | 6 | 1.7 |
| 9 | 150 | 105 | 5 | 8.0 |
| 10 | 190 | 105 | 5 | 9.5 |
| 11 | 170 | 30 | 5 | 8.4 |
| 12 | 170 | 180 | 5 | 10.4 |
| 13 | 170 | 105 | 4 | 9.7 |
| 14 | 170 | 105 | 6 | 13.1 |
| 15 | 170 | 105 | 5 | 12.2 |
| 16 | 170 | 105 | 5 | 12.6 |
| 17 | 170 | 105 | 5 | 11.0 |

Empirical data from Box Behnken experiments is presented in Table A.2. Linear regression models for cellulose yield, kappa number, and pulp viscosity are visualized in Fig. A.1. The final regression model for cellulose yield in Table A.2 was, Eq. (A.2):

$\hat{y}_{ce}=54.3-2.0x_{1}+0.03x_{2}+0.54x_{3}-0.88x_{1}x_{2}+1.28x_{2}x_{3}$ (A.2)

where $\hat{y}_{ce}$ denoted predicted cellulose yield (%) and $x_{1}$, $x_{2}$, and $x_{3}$ the coded values for delignification temperature (^o^C), time (min) and liquid-to-solid ratio, respectively. Observation no. 6 in Table A.2 was excluded from building the model. The values of the coefficients have been rounded.

The final regression model for kappa number yield in Table A.2 was, Eq. (A.3):

${log}_{10}\left( \hat{y}_{k} \right)=0.93-0.61x_{1}-0.16x_{2}+0.13x_{1}^{2}$ (A.3)

where $\hat{y}_{k}$ denoted predicted kappa number and $x_{1}$, and $x_{2}$ the coded values for delignification temperature (^o^C) and time (min), respectively. The values of the coefficients have been rounded.

The final regression model for pulp viscosity in Table A.2 was, Eq. (A.4):

$\hat{y}_{v}=584.7-193.8x_{1}-41.3x_{2}+42.5x_{3}+32.5x_{1}x_{3}$ (A.4)

where $\hat{y}_{v}$ denoted predicted pulp viscosity (ml g^-1^) and $x_{1}$, $x_{2}$, and $x_{3}$ the coded values for delignification temperature (^o^C), time (min) and liquid-to-solid ratio, respectively. The values of the coefficients have been rounded.

Table A.2: The Box Behnken dataset from the experiments.

| Experiment | Temperature (^o^C) | Time (min) | Liquid to solid ratio | Cellulose yield (%) | Kappa number | Viscosity (ml g^-1^) |
| --- | --- | --- | --- | --- | --- | --- |
| 1 | 120 | 60 | 7.5 | 54.9 | 50.3 | 780 |
| 2 | 160 | 60 | 7.5 | 53.2 | 3.6 | 470 |
| 3 | 120 | 180 | 7.5 | 57 | 35.3 | 750 |
| 4 | 160 | 180 | 7.5 | 51.9 | 2.8 | 380 |
| 5 | 120 | 120 | 5 | 56.1 | 61.2 | 780 |
| 6 | 160 | 120 | 5 | 48.8^a^ | 2.4 | 280 |
| 7 | 120 | 120 | 10 | 57.5 | 42.7 | 800 |
| 8 | 160 | 120 | 10 | 52.3 | 2.7 | 430 |
| 9 | 140 | 60 | 5 | 55.4 | 18 | 600 |
| 10 | 140 | 180 | 5 | 52.6 | 5.8 | 500 |
| 11 | 140 | 60 | 10 | 54 | 17 | 690 |
| 12 | 140 | 180 | 10 | 56.3 | 5.3 | 580 |
| 13 | 140 | 120 | 7.5 | 54.4 | 8.9 | 610 |
| 14 | 140 | 120 | 7.5 | 53.2 | 5.9 | 570 |
| 15 | 140 | 120 | 7.5 | 53.2 | 6.5 | 550 |

^a^ Experiment no. 6 was excluded from the cellulose yield model, Eq. (A.2).


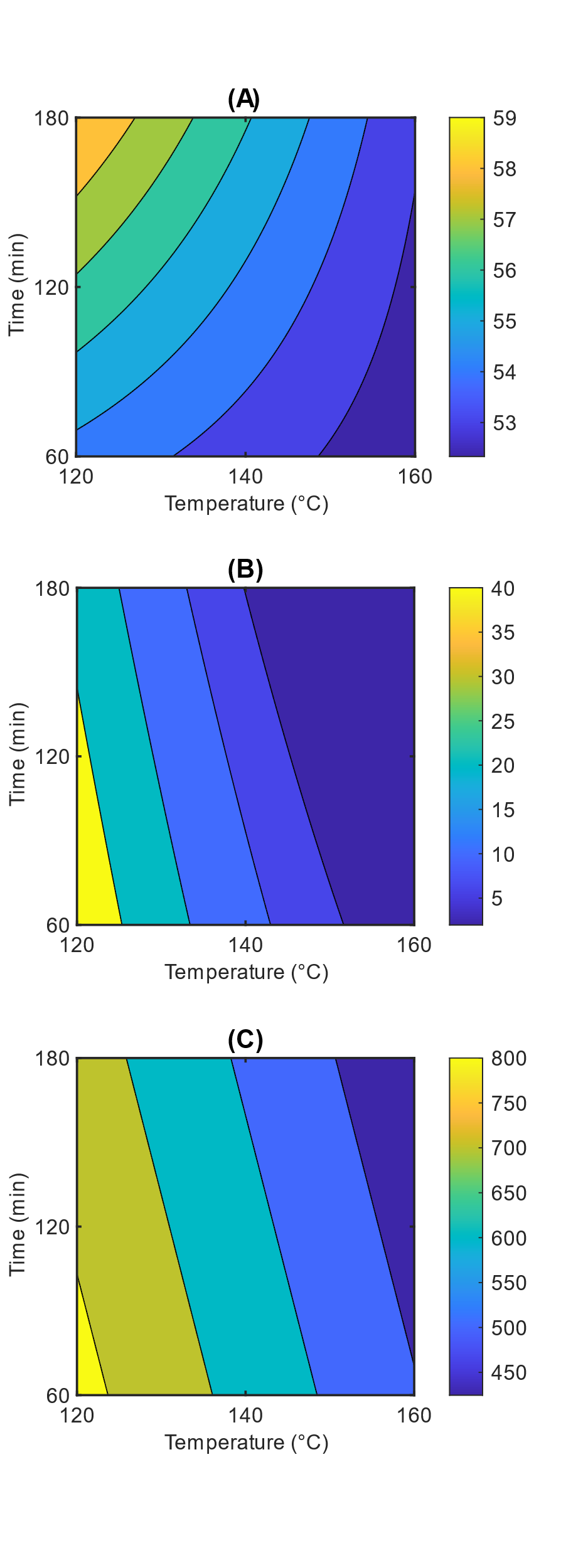


Fig. A.1: Response surfaces of (A) predicted cellulose yield, (B) kappa number and (C) pulp viscosity with a liquid-to-solid ratio of 10.

The empirical data from adaptive Bayesian optimization experiments is presented in Table A.3. Rows 1–5 of Table A.3 were originally Box Behnken experiments 12, 10, 6, 3 and 13 in Table A.2, which were selected a priori for the initialization of the Bayesian optimization algorithm.

The prior distributions for the hyperparameters of Gaussian process regression were set up for the simulated carbohydrate yield experiments, and the same prior distributions were reused in the birch delignification experiments.

On each iteration of Bayesian optimization, the variables and measured objective values were standardized before Gaussian process regression as follows. The variables were scaled and translated to make their feasible range equivalent to $[0,1]$. The measured objective function values were scaled and translated to make their mean 0 and sample standard deviation 1.

The prior distribution for noise standard deviation was a Gamma distribution with shape 2 and rate 2, truncated at 10^-4^ to provide a hard lower bound for the measurement noise. The prior distribution for the length scale parameter of each variable was a smoothed box distribution on [0.2, 1.0] with smoothing parameter 0.1, truncated at a lower bound of 0.1. The probability density function of a smoothed box distribution is of the form, Eq. (A.5):

$f_{\mathrm{sb}}\left( x \right)=\frac{1}{C\left( a,b,\sigma\right)}\exp\left( -\left( \max\left( 0,\frac{a-x}{\sigma},\frac{x-b}{\sigma} \right) \right)^{2} \right)$ (A.5)

where $[a,b]$ is the range of maximum density, $\sigma$ is the smoothing parameter and $C$ is a normalization constant.

Table A.3: The Bayesian optimization dataset from the experiments.

| Experiment | Temperature (^o^C) | Time (min) | Liquid to solid ratio | Cellulose yield (%) | Kappa number | Viscosity (ml g^-1^) | Objective function value |
| --- | --- | --- | --- | --- | --- | --- | --- |
| 1 | 140 | 180 | 10 | 56.3 | 5.3 | 580 | 56.3 |
| 2 | 140 | 180 | 5 | 52.6 | 5.8 | 500 | 52.6 |
| 3 | 160 | 120 | 5 | 48.8 | 2.4 | 280 | -71.2 |
| 4 | 120 | 180 | 7.5 | 57.0 | 35.3 | 750 | -9.3 |
| 5 | 140 | 120 | 7.5 | 54.4 | 8.9 | 610 | 52.9 |
| 6 | 134 | 180 | 7.81 | 54.7 | 7.0 | 560 | 54.6 |
| 7 | 135 | 81 | 10 | 53.4 | 21.3 | 710 | 19.1 |
| 8 | 135 | 126 | 5 | 53.5 | 10.8 | 480 | 51.3 |
| 9 | 139 | 180 | 7.37 | 53.3 | 4.4 | 480 | 53.3 |
| 10 | 134 | 180 | 5 | 53.0 | 6.8 | 460 | 53.0 |
| 11 | 137 | 150 | 6.79 | 53.6 | 6.0 | 480 | 53.6 |
| 12 | 145 | 180 | 10 | 54.3 | 3.4 | 490 | 54.3 |
| 13 | 143 | 133 | 10 | 54.3 | 4.1 | 550 | 54.3 |
| 14 | 143 | 164 | 8.5 | 53.4 | 3.6 | 550 | 53.4 |
| 15 | 142 | 168 | 9.72 | 54.5 | 3.3 | 500 | 54.5 |


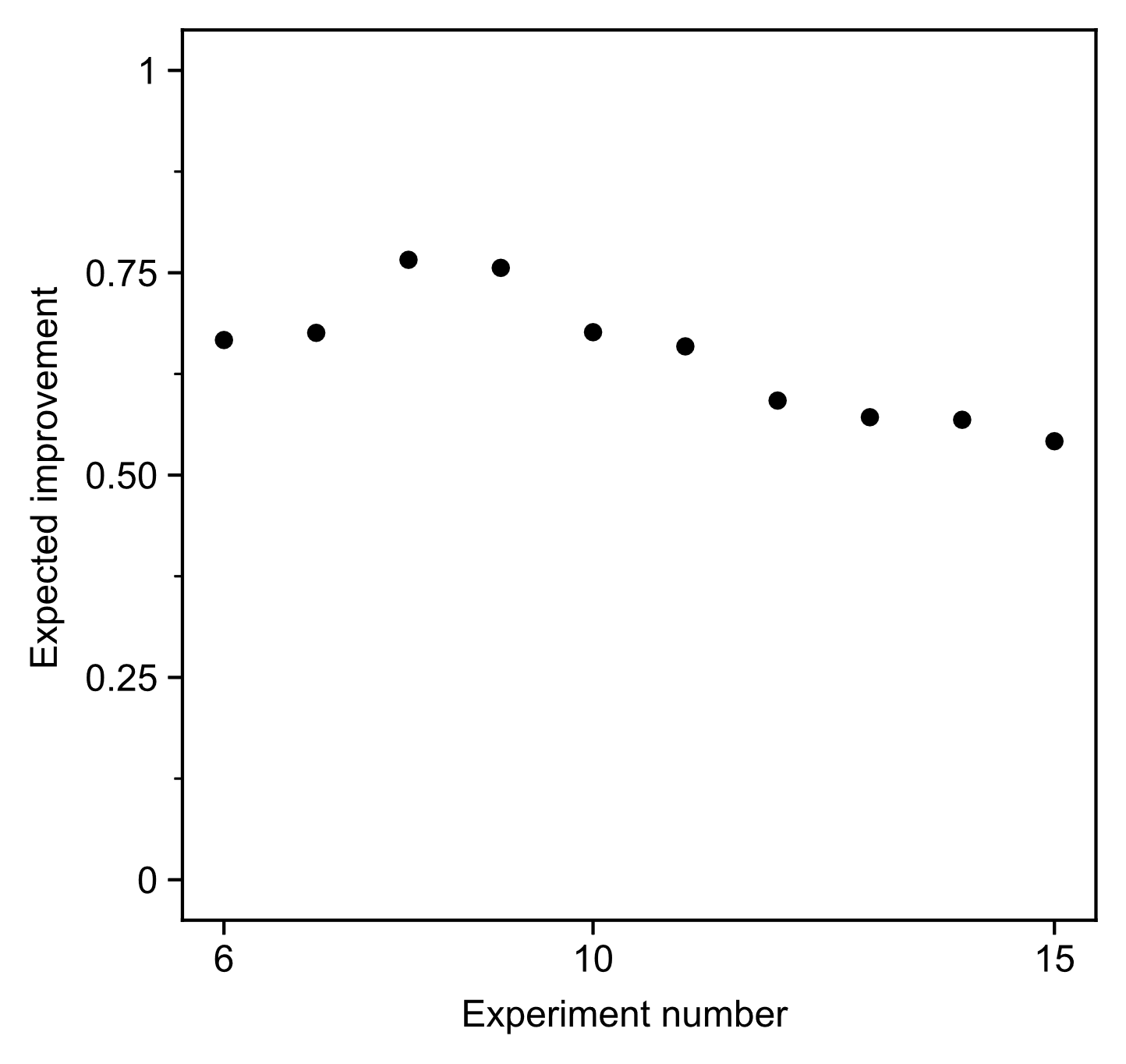


Fig. A.2: The expected improvement in the Bayesian optimization objective (dimensionless), computed before each adaptive cellulose yield experiment by the Bayesian optimization algorithm.


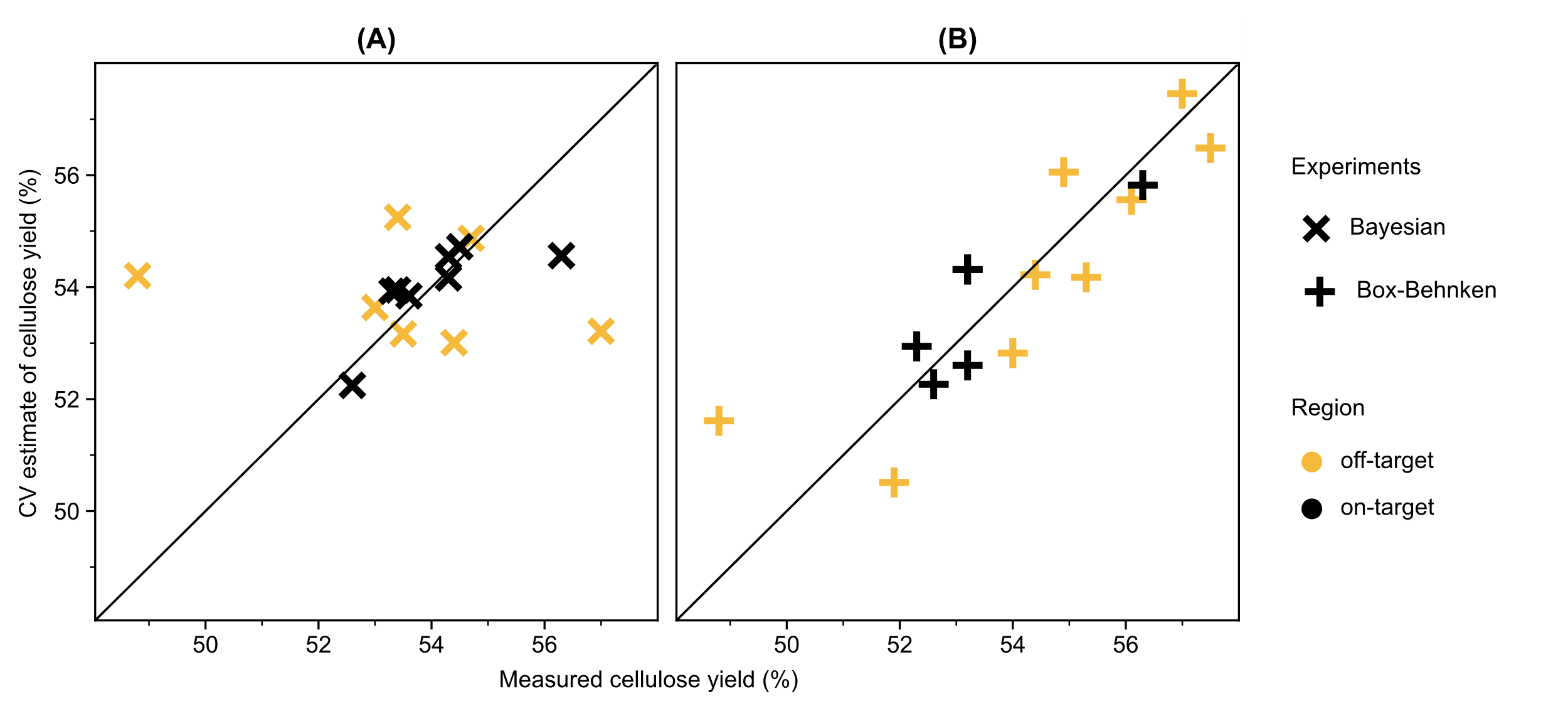


Fig. A.3: Measured cellulose yield versus the predicted cellulose yield from leave-one-out cross-validation: (A) predictions by Gaussian process regression based on Bayesian optimization experiments, (B) predictions by linear regression based on Box Behnken experiments. Black indicates the experiments where kappa number and pulp viscosity were in the target ranges defined in Table 1.
